# Dose rate dependent reduction in chromatin accessibility at transcriptional start sites long time after exposure to gamma radiation

**DOI:** 10.1080/15592294.2023.2193936

**Published:** 2023-03-27

**Authors:** Hildegunn Dahl, Jarle Ballangby, Torstein Tengs, Marcin W. Wojewodzic, Dag M. Eide, Dag Anders Brede, Anne Graupner, Nur Duale, Ann-Karin Olsen

**Affiliations:** a Division of Climate and Environmental Health, Department of Chemical Toxicology, Norwegian Institute of Public Health, Oslo, Norway; b Centre for Environmental Radiation (CERAD), Norwegian University of Life Sciences (NMBU), Ås, Norway; c Division for Aquaculture, Department of breeding and genetics, Nofima, Ås, Norway; d Department of Research, Section Molecular Epidemiology and Infections, Cancer Registry of Norway, Oslo, Norway; e Faculty of Environmental Sciences and Natural Resource Management (MINA), Norwegian University of Life Sciences (NMBU), Ås, Norway

**Keywords:** Gamma radiation, chronic exposure, acute exposure, low dose rate, long-term response, chromatin accessibility, ATAC-Seq, CBA mice strain

## Abstract

Ionizing radiation (IR) impact cellular and molecular processes that require chromatin remodelling relevant for cellular integrity. However, the cellular implications of ionizing radiation (IR) delivered per time unit (dose rate) are still debated. This study investigates whether the dose rate is relevant for inflicting changes to the epigenome, represented by chromatin accessibility, or whether it is the total dose that is decisive. CBA/CaOlaHsd mice were whole-body exposed to either chronic low dose rate (2.5 mGy/h for 54 d) or the higher dose rates (10 mGy/h for 14 d and 100 mGy/h for 30 h) of gamma radiation (^60^Co, total dose: 3 Gy). Chromatin accessibility was analysed in liver tissue samples using Assay for Transposase-Accessible Chromatin with high-throughput sequencing (ATAC-Seq), both one day after and over three months post-radiation (>100 d). The results show that the dose rate contributes to radiation-induced epigenomic changes in the liver at both sampling timepoints. Interestingly, chronic low dose rate exposure to a high total dose (3 Gy) did not inflict long-term changes to the epigenome. In contrast to the acute high dose rate given to the same total dose, reduced accessibility at transcriptional start sites (TSS) was identified in genes relevant for the DNA damage response and transcriptional activity. Our findings link dose rate to essential biological mechanisms that could be relevant for understanding long-term changes after ionizing radiation exposure. However, future studies are needed to comprehend the biological consequence of these findings.

## Introduction

Ionizing radiation (IR) is an environmental carcinogen [1], with natural (radon, cosmic, soil and food) and human-made (medical, nuclear industry and power plant accidents) exposure sources. Exposure to IR occurs through different radiation regimes (low or high doses and dose rates; acutely, chronically, or protracted). Solid cancers [2] and leukaemia [3] are well-known radiation-induced human health effects [4]. However, health effects also extend to other possible conditions, including cardiovascular [5–7], metabolic [8,9] and ocular diseases [10]. The predictions of health effects from exposure to IR are based on populations mainly exposed to high doses and high dose rates (e.g., A-bomb survivors from Hiroshima and Nagasaki (the Life Span Study) [11]). Whether the risk coefficients drawn from these studies are relevant when predicting health risks from nuclear incidents where lower doses and dose rates of IR are more typical, like the Chernobyl [12] and the Fukushima Daiichi nuclear powerplant accidents [13], is still debated [14–16].

Ionizing radiation introduces a range of cellular effects, from direct DNA damage and the induction of reactive oxygen species (ROS) [17,18]. These IR-induced insults further activate events to restore cellular and genetic integrity, like the recognition of DNA damage, cell cycle arrest, damage repair, and cellular death [19–21]. Events dependent upon the dynamic regulation of the chromatin structure [22–25]. Epigenetic changes are also reported after radiation exposure [26–28], like DNA methylation of cytosines [29] and post-translational modification of histones [30–32]. These epigenetic mechanisms can adopt chromatin accessibility without changing the DNA sequence. The chromatin can thus be viewed as the functional form of genetic information referred to as the epigenome. Gene expression and transcriptional activity are, therefore, intimately linked to the chromatin structure and the remodelling dynamics [33]. Over the years, studies have addressed altered gene expression as a mechanistic explanation for radiation-induced outcomes [34–37]. However, how these responses progress to disease and how the dose rate is relevant to the outcome is debated [38–42].

There is a growing understanding of the epigenome’s relevance for cancer initiation and progression [43]. Therefore, considering the extent of IR-induced responses affecting the epigenome, mapping the radiation-induced changes in chromatin accessibility (how it rearranges upon exposure and how this could be linked to changes in gene expression) could be essential for establishing causality between radiation-induced effects and the progression of adverse health effects. The Assay for Transposase-Accessible Chromatin using DNA sequencing (ATAC-Seq) is an epigenomic method for mapping open chromatin regions (OCRs) using a probing transposase (Tn5) [44,45]. The Tn5 cleaves the DNA at open chromatin regions and simultaneously inserts adapters for high-throughput DNA sequencing (HTS). The Omni-ATAC-Seq [46] reduces the contamination from mitochondrial DNA and is optimized for frozen tissues making it suitable for extensive animal experiments.

In this study, we investigated two hypotheses related to the epigenomic effects of ionizing radiation. We hypothesize that exposure to gamma radiation inflicts significant changes in the epigenomic feature of chromatin accessibility. Furthermore, we hypothesize that radiation-induced changes in chromatin structure persist over time, depending on the dose rate. The hypotheses were addressed by characterizing whole-genome chromatin accessibility in liver tissue of mice exposed to acute high, intermediate, or chronic low dose rate gamma radiation, all to a total dose of 3 Gy.

## Materials and methods

### Animals and housing

This study follows the previous descriptions of the animal experiment [36,47]. Specific Pathogen Free CBA/CaOlaHsd (3–8 weeks) mice were purchased from Envigo (Horst, The Netherlands). Acclimation took place for a minimum of 4 d. The mice were then randomly housed in groups of two to three in individually ventilated disposable PET plastic cages (IVC racks) (Innovive, San Diego, USA) using Aspen tree bedding (Nestpack, Datesand Ltd., Manchester, UK). Temperature and light conditions were controlled (21 ± 2°C, 45 ± 15% relative humidity, 50 air changes h^−1^ and photoperiod 12:12 (L:D). Mice had ad libitum access to tap water in PET bottles and SDS RM1 feed (Special Diet Services, Essex, UK). Due to space limitations in the radiation field, the mid-dose rate (MDR) groups were housed outside the IVC rack during irradiation, but in the same disposable PET cages but using transport lids. At termination, the mice were administered anaesthesia using ZRF-cocktail (Zolazepam, Tiletamine, Xylazine, and Fentanyl) followed by heart puncture before cervical dislocation and collection of tissues. The tissues were snap-frozen in liquid nitrogen and stored at −80°C. We adhered to the national legislation for animal experimentation, and the experimental protocol was approved by the Norwegian Food Safety Authority (NFSA, approval no. 8803). No mice died or showed clinical signs due to the exposure.

### Radiation and dosimetry

**Figure 1.**
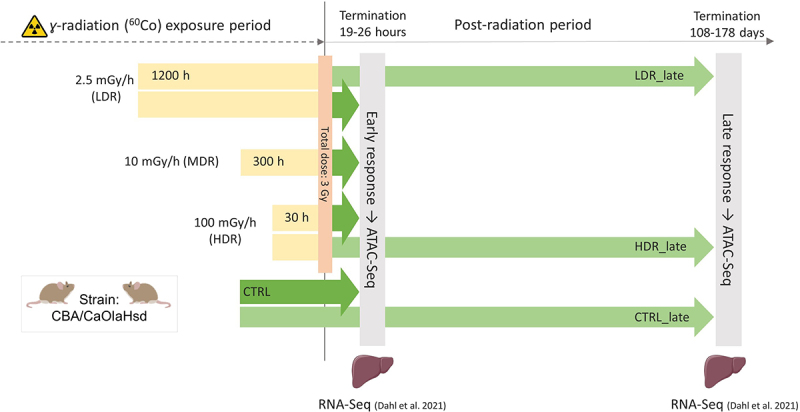
*Experimental design* ATAC-Sequencing was utilised to investigate radiation-induced effects on liver chromatin accessibility at two post-radiation timepoints: early (19–26 hours) and late (108–178 d). Gamma radiation was administered using three dose rates (low (LDR), mid (MDR) and high HDR)) to a total dose of 3 Gy. Liver samples were collected for ATAC-Seq (current study) and transcriptional response both early and late (RNA-Seq) [36]. .

As previously described [47], all groups received gamma radiation (^60^Co-source) exposure using different dose rates (DR); 2.5 (low DR (LDR)), 10.0 (mid DR (MDR)) and 100.0 (high DR (HDR)) mGy/h (Table 1, Figure 1). The pre-calculated duration of exposure was 1200 h, 300 h and 30 h for the respective groups. Dosimetry was performed using nanoDots as described [48,49]. The numeric value of air kerma to whole-body absorbed dose conversion coefficient for chronic exposures was 0.932 ± 0.008, resulting in a total whole-body absorbed dose of 2.60 ± 0.19 Gy for the 2.5 mGy/h-group, 2.67 ± 0.16 Gy for the 10 mGy/h-group, and 2.65 ± 0.13 Gy for the 100 mGy/h-group, all denoted as 3 Gy throughout the article. The irradiation took place at the FIGARO low dose gamma irradiation facility, managed by the CoE Centre of Environmental Radioactivity (CERAD CoE, Norwegian University of Life Sciences, Ås, Norway) [48,50,51]. For animal care, the irradiation was interrupted daily (30–120 min). Thus, the beam-on time was adjusted according to animal care off-time to achieve the pre-calculated total dose of 3 Gy. Cage positions were rotated daily to assure uniform exposure. Unexposed control mice were housed behind lead shielding outside the radiation field but inside the exposure room. Further details regarding dosimetry and the current experimental design have been described [36,47].

**Table 1.** Experimental descriptive details: groups, post-radiation time in days, dose rate, age at termination in days, and number of samples per group used in ATAC-Seq.

	Groups	Days post-radiation mean ± SD	Nominal dose rate (mGy/h)	Whole-body absorbed dose (Gy)	Age at termination (days) mean ± SD (range)	n samples ATAC-Seq
Early response	CTRL	-	-	-	70 ± 0	5
	LDR	1 ± 0	2.5	2.60 ± 0.19	112 ± 0	3
	MDR	1 ± 0	10	2.67 ± 0.16	70 ± 0	3
	HDR	1 ± 0	100	2.65 ± 0.13	63 ± 0	3
Late response	CTRL_late	-	-	-	185 ± 70 (118–245)	4
	LDR_late	108 ± 0	2.5	2.60 ± 0.19	216 ± 0	3
	HDR_late	149 ± 35	100	2.65 ± 0.13	248 ± 34 (216–284)	3

### Experimental design

The mice (*n* = 35) were divided into four experimental exposure groups (controls (CTRL), LDR, MDR and HDR) (Figure 1, Table 1). The CTRL, LDR, and HDR were further divided into two post-radiation termination groups. A total of seven experimental groups were thus generated; four groups were terminated 19–26 hours after the end of irradiation (Early response groups: CTRL, LDR, MDR and HDR, and three groups were terminated after a post-radiation period of 108–178 d (late response groups, CTRL_late, LDR_late, and HDR_late. CTRL and CTRL_late represent the corresponding control groups for the two termination timepoints, early and late, respectively. The mice were transported to the Norwegian Institute of Public Health (NIPH, Oslo, Norway) for post-radiation housing until termination.

### Assay for transposase-accessible chromatin (ATAC-Seq)

Samples (*N* = 24) were processed using an ATAC-Seq protocol for frozen tissues (Omni-ATAC) [46]. Approximately 20 mg of liver tissue was collected from snap-frozen samples stored at −80°C and homogenized in 1 mL OptiPrep solution D in a 7 mL Kimble Dounce tissue grinder set (DWK Life Sciences, Mainz, Germany) as described [46]. All steps were performed on ice unless specified otherwise. The tissue was homogenized using six strokes with pestle A and six with pestle B. The homogenate (400 µL) was diluted 1:1 in OptiPrep solution C (5:1 of OptiPrep solution A and OptiPrep solution B (Sigma-Aldrich® Brand (cat. nr: D1556), Merck KGaA, Darmstadt, Germany)) to a final concentration of 25% iodixanol. A gradient consisting of two layers of iodixanol, 29% (w/v) and 35% (w/v), was used to separate the nuclei (3000 × g, 4°C, 20 minutes). The band of nuclei was extracted (200 µl) and diluted in 800 µL ATAC-RSB, and pelleted at 500 × g for 10 min at 4°C. The nuclei pellet was suspended in ATAC TD-buffer (22 mM Tris – HCl pH 7.4, 10 mM MgCl2, 20% Dimethylformamide; pH 7.4) to a final concentration of 50 K − 100 K nuclei/50 µL. The nuclei solutions (50 µL) were incubated at 37°C for 30 min with 2.5 μL Illumina Tagment DNA Enzyme Illumina, San Diego, CA, USA (cat. nr: 20034197) for tagmentation. The fragmented DNA was purified using PCR Purification Kit (QIAGEN, Hilden, Germany) and eluted in 20 µL elution buffer. Amplification of the tagmented DNA (20 µL) was performed using 25 µL 2× NEBnext High-Fidelity PCR Master Mix (New England BioLabs (cat. nr: M0541L), Ipswich, MA, USA) and 2.5 µL forward and reverse Nextera DNA CD indexes (Illumina, San Diego, CA, USA). The cycling conditions were: (1) 72°C for 5 min, (2) 98°C for 30 sec, (3) 98°C for 10 sec, (4) 63°C for 30 sec, and (5) 72°C for 30 sec. Steps 3–5 were repeated five times. Based on a tape station trace (4200 TapeStation, Agilent, Santa Clara, USA), the libraries were further amplified with 5–7 cycles (to a total of 10–13 cycles). The libraries were purified and size-selected using AMPure XP beads (Beckman Coulter, Brea, CA, USA) to eliminate fragments <100 nt and >1500 nt and diluted to 5 nM. Paired-end sequencing (PE150) with an average depth of 50 million raw reads was sequenced on Illumina NovaSeq6000 at Novogene Co., Ltd (Cambridge, UK)).

### Pre-processing of sequencing reads and downstream analysis

#### Sequencing

The exact parameters of pipelines used for raw-data and differential analysis are presented in Supplementary_1 (S1). The FASTQC files were quality controlled using the FASTQC tool (bioinformatics.babraham.ac.uk/projects/fastqc/). Adapter trimming was performed using Trim Galore! (bioinformatics.babraham.ac.uk/projects/trim_galore/). The reads were aligned to the mouse genome (GRCm38) with BWA [52] using the nf-core ATAC-seq pipeline [53]. Further, the reads from accessible regions (<100 nt) were extracted from the Binary Alignment Map (BAM) files and peak called using MACS2 (v2.2.7) [54]. The quality of peaks were controlled using the ChIPQC (v1.26.0) [55], and non-overlapping consensus peaks in 8 of the 24 biological samples were used for differential analysis. The ATAC-Seq raw reads supporting the findings in this study are made openly available at the public NCBI Sequence Read Archive (SRA) (www.ncbi.nlm.nih.gov/sra), using BioProject accession id: PRJNA832920.

### Analysis of differentially accessible regions (DARs)

All downstream analysis was performed using R-statistical environment (R-Core Team (2020)). Differentially accessible peaks were called using DESeq2 (v1.30.1) [56] and adjusted for the age of animals, as age could potentially introduce changes in chromatin structure [57]. Statistically significant differentially accessible regions (DARs) were identified using a false discovery rate (FDR) <0.1 when comparing exposure groups to the respective control group (early: LDR vs CTRL, MDR vs CTRL, HDR vs CTRL and late: LDR_late vs CTRL_late and HDR_late vs CTRL_late). DAR-associated genes (DAGs) were annotated using ChIPseeker (v1.26.2) [58] with ‘org.Mm.eg.db’ (3.8.2) [59]. Entrez gene identifiers were used. All the genes in proximity to the DARs (regardless of distance and genomic region) were identified as a DAR-associated gene (DAG) (‘nearest approach’).

### Enrichment analysis of DAR-associated genes (DAGs)

MetaScape (v3.5, used: 23.02.2022), a web-based tool, (http://metascape.org) was used for multiple gene-lists enrichment analysis [60]. In short, the default settings for enrichment were used and covered the following ontology sources: KEGG Pathway, GO Biological Processes, GO Cellular Components, GO Molecular Functions, Reactome Gene Sets, CORUM, TRRUST, PaGenBase, WikiPathways and PANTHER Pathway. P-value <0.01 (accumulative hypergeometric distribution), min. overlap of three genes, and an enrichment factor >1.5 were used to identify statistically significant terms. By defult, the whole genome is used as background gene list by MetaScape for enrichment analysis. The top 20 statistically significant terms represent each cluster in the cytoscape, surrounded by membership terms with a similarity score >0.3. Benjamini-Hochberg procedure is used for adjusted p-value (q-value). The complete MetaScape-output is found in Supplemantary_2 (S2).

### Comparing the ATAC-Seq data with RNA-Seq

The association between differentially expressed genes (DEGs) from our previously reported RNA-Seq data [36] and the DAGs in the current study was performed. Hepatic RNA isolation, mRNA sequencing and data analysis are described in [36]. The list of differentially expressed genes (DEGs) were found from in Dahl et al. (2021) supplementarytable 1, and cross-analysed with the ATAC-Seq data. The RNA sequencing raw data is available at the NCBI Sequence Read Archive (SRA) (PRJNA747753).

First, the DAGs overlapping a DEGs were visualized using the web-based tool, InteractiVenn [61]. Further, the mRNA expression levels (log_2_FC) were categorized as ‘up-regulated’ or ‘down-regulated’ based on the expression level threshold cut-offs of log_2_(FoldChange)>±0.3, respectively. When the mRNA log_2_(FoldChange) fell between the cut-offs, the mRNA expression was classified as ‘stable’ (unaffected by exposure). If the gene identified as a DAG had no mRNA data, the DAG were classified as ‘not expressed.’ The DAGs were grouped based on the chromatin accessibility as reduced (negative log_2_-ratio) or gained (positive log_2_-ratio), and the two categories presented in a mosaic plot.

## Results

To investigate the influence of dose rate (chronic and acute) on chromatin landscape, whole-genome ATAC-Sequencing were performed on tissue collected from mouse livers. The chromatin accessibility was evaluated at two post-radiation timepoints: one day post-radiation (early) and after a longer post-radiation period (late). We will focus on the LDR and HDR exposure groups, both early and late.

### Quality control of ATAC-Seq

**Figure 2.**
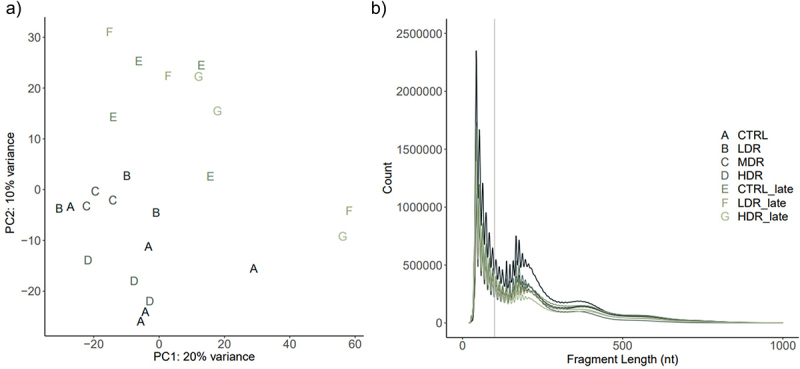
*ATAC-Seq quality control by the sequenced fragments length distribution and principal component analysis* a) a principal component analysis (PCA) plot illustrates the samples sorted represented by experimental group using upper-case letter and color. b) Transposase tagmentation sequence fragment lengths distribution. Each line represents the mean counts per fragment per experimental group. Fragment lengths up to 100 bp represents the ATAC-Seq fragments corresponding to nucleosome-free regions (NFRs) used for peak calling. The characteristic shape of waves along the x-axis (fragments length) represents fragments spanning nucleosomes; mono- (186–282 bp), di- (ca 400 bp) and tri- (ca 600 bp) nucleosomes. The fragment distribution per sample in Supplementary 4 (S4).

The quality of the libraries was assessed both to validate the ATAC-Seq protocol and the results according to recommendation [44,46]. The tagmentation procedure showed the expected distribution with abundance of sequenced fragments less than 100 bases and progressively fewer fragments of larger size (Figure 2a). Principal component analysis (PCA) showed no batch effects (Figure 2b). After quality filtering and adaptor removal, the overall rates of aligned reads to mg38 ranged from 93.8% to 98.6% (Supplementary_3 (S3)). A total of 65,981 consensus peaks were identified when merging the peak data sets from controls and experimental groups. The identified peaks were associated with 31,121 ENSMUST transcripts, representing 17,164 different genes (Ensembl 102) [62].

### Radiation-induced changes in genome-wide chromatin accessibility

**Figure 3.**
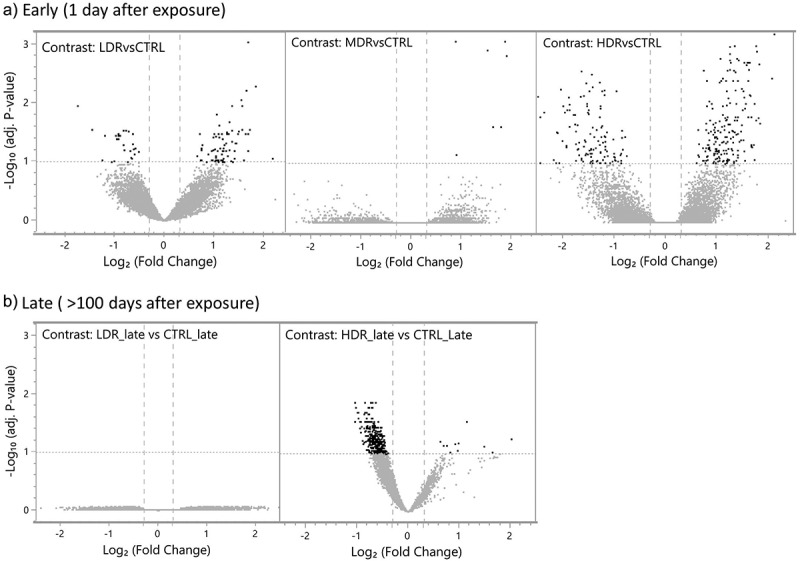
*Differentially changed accessible regions (DARs)* the statistical significance and the magnitude of the differentially changed accessible regions (DARs, dark spots) are presented as repressed (upper left quadrant) or gained (upper right quadrant) compared to controls, using false discovery rate (FDR) <0.1, illustrated by the horizontal line at -log.

To identify changes in the chromatin landscape driven by dose rate at the post-radiation timepoints, we contrasted all exposure groups to their respective control group. Statistically significant (FDR < 0.1) differentially accessible regions (DARs) were identified for all contrasts except LDR_late vs CTRL_late (Table 2, Figure 3). The magnitude of changed chromatin accessibility and the statistical significance is illustrated in volcano plots (Figure 3). Higher numbers of DARs were observed in HDR compared to LDR one day post-radiation (early) (Table 2). By stratifying the early response DARs in gained accessibility (positive log_2_-ratio) and reduced accessibility (negative log_2_-ratio) more than 60% of the DARs in all three groups showed gained accessibility compared to control (CTRL). Few DARs were identified for MDR, all of them had gained accessibility.

**Table 2.** *Differentially accessible regions (DARs) by dose rate.* The DARs (FDR < 0.1) are stratified into regions with gained or reduced accessibility contrasted to the respective control group (relative percentage in brackets). The total number of DAR-associated genes (DAGs) is listed with the numbers of DAGs also identified by RNA sequencing in Dahl et al. 2021 in brackets (*mrna)).

	Group Contrasts	Total DARs	Reduced accessibility	Gained accessibility	DAGs (*mRNA)
Early response	LDR vs CTRL	100	26 (26%)	74 (74%)	96 (67)
	MDR vs CTRL	7	0	7 (100%)	7 (6)
	HDR vs CTRL	326	121 (36%)	205 (64%)	295 (161)
Late response	LDR_late vs CTRL_late	0	0	0	0
	HDR_late vs CTRL_late	371	360 (97%)	11 (3%)	364 (331)

Following the longer post-radiation period (late), the chromatin accessibility in chronic low dose rate exposed mice (LDR_late) were not different from control mice, while the high dose rate exposed mice were markedly different from controls (HDR_late). The DARs identified for HDR_late vs CTRL_late demonstrated almost exclusively reduced accessibility (Table 2).

**Figure 4.**
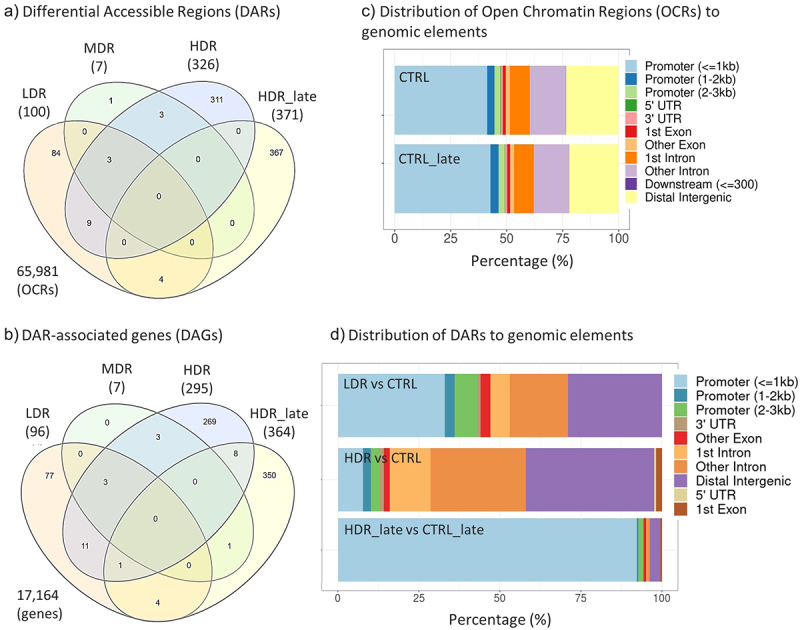
*Overlap of Differential Accessible Regions (DARs) and DAR-associated genes (DAGs), and the allocation of open chromatin regions (OCRs) and DARs to genomic elements.* Venn diagrams of DARs (a) and DAGs (b) for all experimental groups. The numbers of DARs and DAGs for each group in brackets. Total numbers of identified OCRs (a) and the corresponding genes (b) outside Venn diagram. c) Allocation of OCRs to genomic elements for both controls (CTRL and CTRL_late) after merging the biological replicates. d) Allocation of DARs after contrasting the experimental group to respective controls. MDR and LDR_late are not represented in d) due to few or no DARs identified, respectively. The distribution of the DARs within the genomic elements of the exposure groups were tested by χ^*2*^*-test and found statistically significant different (χ*^*2*^*statistics = 472.62, p-value < 0.001).*

The overlap between exposure groups was evaluated to identify a radiation dose-specific chromatin DAR peak signatures. The most prominent finding is the large fraction of dose-rate- and timepoint-specific responses, as only few of the DARs overlapped between the exposure groups. All the early groups share three DARs, LDR_early and HDR_early shared nine DARs, HDR_late shared four DARs with LDR_early. HDR_late did not share any DARs with HDR_early (Figure 4a).

### Functional classification of DARs

The genomic elements containing the open chromatin regions (OCRs) were identified (Figure 4c, Table 3). The results showed that the occupancy within genomic elements differed between dose rates and post-radiation timepoints. At DPR1, DARs were mostly present within promoters (≤1kb), distal intergenic and intronic regions. However, the DARs occurrence differed between chronic LDR and the acute HDR exposure one day post-radiation, which demonstrated fewer DARs in promoter regions and more in intergenic and intronic regions (Supplementary Table). The HDR_late DARs were almost exclusively located in promoter (≤1kb) regions (92%). Of these 94% were found at the transcriptional start site (TSS).

**Table 3.** Relative portion of open chromatin regions (OCRs) and differential accessible regions (DARs) allocated to genomic elements.

Genomic Element	Open Chromatin Regions (OCRs)							Differential accessible regions (DARs)			
	Early				Late			Early			Late
	CTRL	LDR	MDR	HDR	CTRL_late_	LDR_late_	HDR_late_	LDR vs CTRL	MDR vs CRTL	HDR vs CTRL	HDR_late_ vs CTRL_late_
Promoter (<=1kb)	41.3	42.4	43.3	30.9	42.8	49.2	50.8	33.0	28.6	7.7	92.2
Distal Intergenic	23.5	22.5	21.8	26.3	22.1	20.1	19.9	29.0	28.6	39.6	3.2
Other Intron	16.2	15.9	15.5	19.6	15.7	13.8	13.6	18.0	28.6	29.4	0.8
1st Intron	8.8	8.8	9.0	10.7	8.8	7.6	7.3	6.0		12.6	0.3
Promoter (1-2kb)	3.3	3.4	3.6	4.3	3.7	3.3	2.7	3.0		2.5	0.5
Promoter (2-3kb)	2.6	2.6	2.7	3.4	2.7	2.2	1.9	7.0		2.8	1.3
Other Exon	1.9	1.9	1.8	2.0	1.8	1.6	1.6	3.0	14.3	1.8	0.8
1st Exon	1.2	1.3	1.2	1.2	1.2	1.2	1.2			1.8	0.5
3’ UTR	1.0	1.0	1.0	1.3	1.1	0.9	0.8	1.0		1.2	0.3
5’ UTR	0.2	0.1	0.1	0.1	0.1	0.2	0.1			0.6	
Downstream (<=300)	0.1	0.1	0.1	0.1	0.1	0.1	0.1				
Total (%)	100	100	100	100	100	100	100	100	100	100	100

### The DAR-associated genes (DAGs)

**Figure 5.**
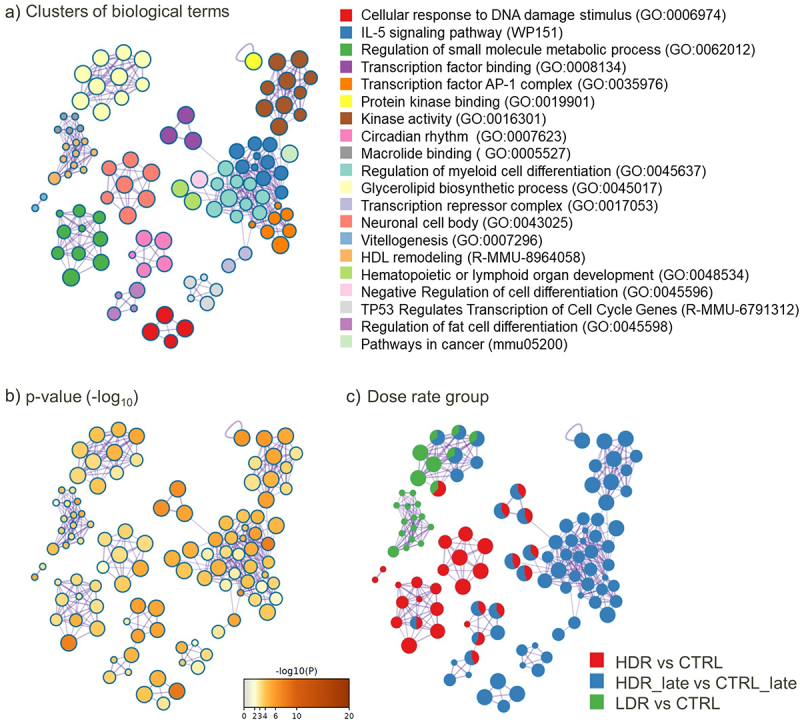
*Network of top 20 enriched biological terms of the DAGs*. the identical clustering network is presented as: (a) biological terms by colouring. Node size reflects number of input genes. The list of terms is sorted by p-value. b) p-value (-log10(p-value)), and c) coloured according to contrast group, where each pie sector is proportional to the number of hits from the respective input gene list. The MDR exposure group is not represented due to few DAGs.

Only few DARs were localized to the same gene, seen by the lowered numbers of DAGs compared to DARs in Table 2. As for the DARs, most of the DAGs are dose-rate-specific (Figure 4b) and a slight increase in the number of overlapping DAGs between the dose rate groups were seen. Even if the gene overlap was marginal, the enriched pathways network showed shared biological functions between HDR_early and HDR_late (Figure 5, Supplementary 2).

In general, the network of enriched pathways also supports a specific response for each dose rate, both early and late. Few significantly enriched pathways were identified for LDR_early and for HDR_early DAGs. The numbers of MDR DAGs were too few to impact the overall enrichment analysis (Supplementary 2). The highest number of enriched terms were found for HDR_late, and they were in majority specific to this group (Figure 5c). Enriched terms found for LDR_early is related to aspects of lipid metabolism (Glycerolipid biosynthesis (adj. p-value 0.009) and HDL remodelling (adj. p-value 0.013)). For HDR_early, the trend was the same, with few enriched pathways, which functions were related to the GO term ‘Small-molecule metabolic processes’ (adj. p-value 0.0004).

The most enriched pathway for HDR_late included ‘Cellular response to DNA damage stimulus’ (adj. p-value 0.0001), ‘IL-5 signalling pathway’ (adj. p-value 0.0001) and ‘Transcription factor AP-1 complex’ (adj. p-value 0.0031). The cluster ‘Cellular response to DNA damage stimulus’ (red nodes in Figure 5A) also comprises the GO term ‘DNA Repair’ and ‘Double-strand break repair’.

HDR_late shared only five terms with HDR_early, although only slightly significant for HDR_early. Examining genes enriched to several terms, mutual DAGs are shared between ‘Transcription factor binding’, ‘Negative regulation of cell differentiation’ and ‘Haematopoietic or lymphoid organ development’. ‘Circadian rhythm’ and ‘Regulation of fat cell differentiation’ also shared DAGs.

### Association between chromatin accessibility and transcription profile

**Figure 6.**
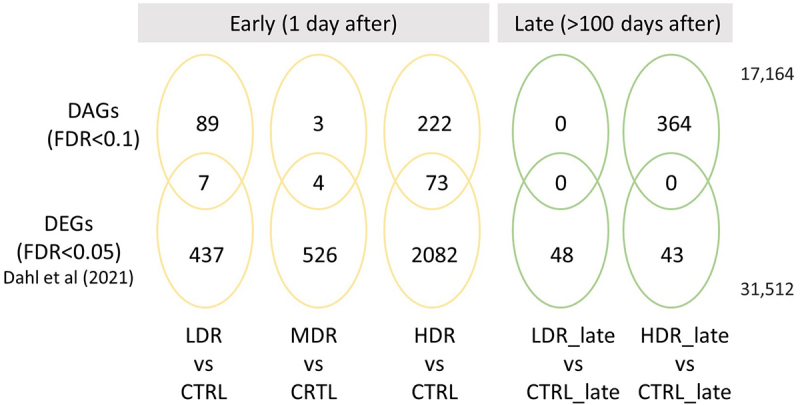
*The association between the DAGs and previously reported differentially expressed genes (DEGs)* Overlap between statistically significant DAGs (Fdr<0.1) derived from ATAC-Seq and the previously published statistically significantly (Fdr<0.05) differentially expressed genes (DEGs) in Table S1 found in Dahl et al (2021) [*36]. Total numbers of genes outside Venn diagram.*

**Figure 7.**
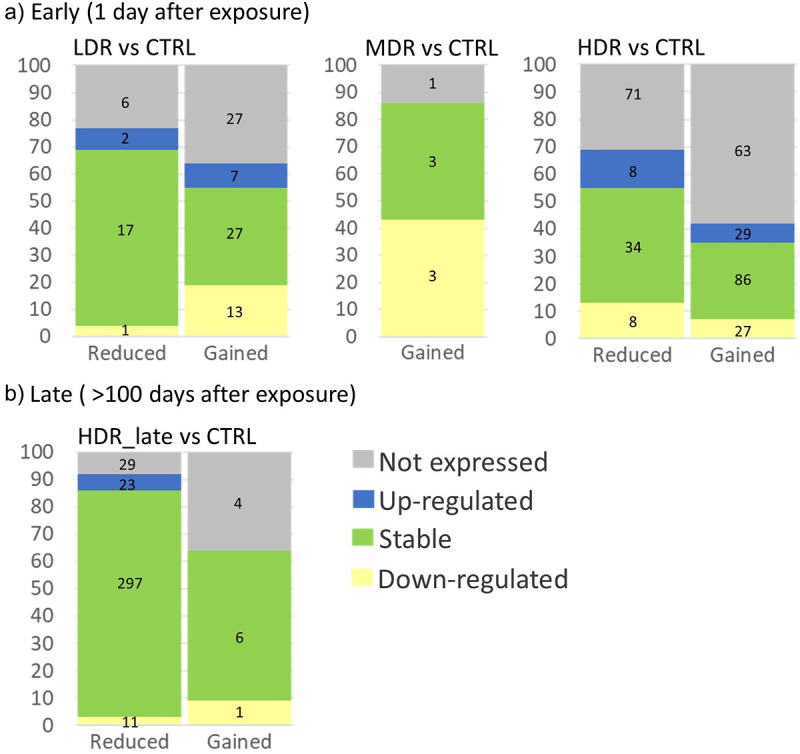
*The association between the chromatin accessibility and the gene expression directions for each exposure group a) early (1 day after) and b) late (>100 d after).* Each DAR corresponds to the nearest gene (DAGs), and the expression of these DAGs have been extracted using the RNA-Seq data [*36]. The DAGs expression level is categorised as “not expressed” (not detected mRNA), “up-regulated” when log*_*2*_*(FoldChange)>0.3, “stable” when −0.3≤log*_*2*_*(FoldChange)≤ 0.3) and “down-regulated” when Log*_*2*_*(FoldChange)<-0.3). The mosaic plots represent the percentage of genes in each RNA expression category, and the numbers inside the bar show the number of genes in each category. The mosaic plot for LDR_late is missing as no DARs were identified*. .

Chromatin structure is relevant for gene expression, and the overlap between the identified DAGs and the differentially expressed genes (DEGs) [36] was explored (Figure 6). Some overlap between the early DAGs and the DEG was seen. Further on, no overlap was observed late after exposure. However, correlating the DAGs and the RNA-Seq gene transcripts, only very weak correlations were seen between these variables (data not shown). Therefore, the association between the DARs chromatin state (reduced accessibility, gained accessibility, or stable) and the direction of gene expression (down-regulated, up-regulated, stable, or not expressed) are addressed in Figure 7.

Most of the identified DAGs (regardless of chromatin state) had stable RNA transcription levels, and no clear association were seen between a reduction or increase in the accessibility and the expression level of the cognate gene. A higher portion of the DAGs identified early, compared to late, had no detectable transcripts. Even if the DARs for HDR_late showed reduced accessibility, most of the cognate genes showed stable gene expression levels (297), whereas a small fraction of the genes was up-regulated (23), and another fraction was down-regulated (11).

## Discussion

This study explores how chronic low dose rate gamma radiation impacts chromatin accessibility and whether changes in chromatin could persist long time after exposure to ionizing radiation. We demonstrate that modifications to the epigenome, represented by chromatin accessibility, were dose rate dependent, not only one day post-radiation but also after a post-radiation period of more than 3 months. We found that exposure to chronic low dose rate; 1) generated a different chromatin pattern compared to acute high dose rate one day post-radiation, and 2) the chromatin state was restored and comparable to controls over 3 months after irradiation. Long-term chromatin changes were only observed after acute HDR exposure. These changes were evident by the reduction in chromatin accessibility at transcriptional start sites (TSS) of genes related to DNA double-strand breaks and regulation of transcriptional activity.

We have focused on the LDR and HDR-groups, both early and late, since few DARs were identified in the MDR_early group, and no linear dose-rate dependent trend was observed. Similar findings for the MDR group were also observed in our previous RNA-Seq data [36]. One possible explanation for the observed MDR group discrepancy might be related to the space limitations in the radiation exposure field, resulting in housing of the MDR mice in cages outside the IVC rack. However, non-monotonic dose responses are previously observed and discussed by others [28,63,64], alleging that the reduced response for a mid-dose rate could be linked to biological aspects rather than experimental issues, and should be pursued in future studies.

One day post-radiation, the results support that gamma radiation remodels chromatin accessibility. This remodelling appeared dose-rate-specific, where HDR exposure led to more extensive changes than LDR exposure. These results are coherent with the transcriptomic profiles that also demonstrated gene perturbations to be dose-rate-specific [36]. This pattern was seen for DARs, DAGs and the DAGs functional enrichment analysis. Common traits between LDR and HDR were seen when the allocation of the DARs to the genomic elements, where the most striking difference was found in intergenic and intronic regions where the number of DARs allocated to the regions increased with dose rate. Except for MDR, which showed few chromatin changes. Intergenic and intronic regions are assumed to possess essential transcriptional regulatory regions like enhancer elements [65]. Most of the identified DARs gained accessibility (>60%) one day post-radiation for both the LDR and HDR exposure, indicative of a possible linkage to the increase in transcriptional activity [36].

The enrichment analysis of both LDR_early and HDR_early DAGs revealed terms related to metabolic processes. Mechanisms previously showed to respond to radiation [66,67]. Further, the enrichment analysis revealed few statistically significant terms for both LDR_early and HDR_early. Since all the DARs were cognate to the nearest proximal gene, this could introduce ‘false genes’ and confound the enrichment analysis. Implying that the DARs harbour distal regulatory properties to other genes than the nearest. The high number of identified DAGs where the mRNA transcript is not expressed [36] could also support this (Figure 7).

As the ATAC-sequencing method is based on the depletion of nucleosomes, it is presumed that the mapped reads should be in regions associated with transcriptional activity. However, comparing the DARs with the DEGs from the RNA-Seq analysis [36], some overlap is seen one day post-radiation and none later. Studies attempting to correlate gene expression data with ATAC-Seq data are inconsistent. Some studies show correlation between the chromatin state and gene expression [68–72], and others report no correlation [73,74]. Due to this, and the lack of observed correlation between the data sets herein (data not shown), we stratified the DARs and associated them with mRNA stratified on expression direction (Figure 7). This exercise demonstrated that chromatin regions with reduced accessibility were proximal to or within upregulated genes and vice versa. This indicates that changes in chromatin configuration are not strongly associated with mRNA expression in samples collected at the same time points (Figure 7), as also observed by others [68]. We assume that the transcriptional activity observed could represents past events compared to the chromatin status. Hence, differentially expressed genes do not necessarily have differentially changed accessible regions nearby in response to radiation. It should be noted that reduced chromatin accessibility also can be linked to increased transcriptional activity through binding of regulatory proteins [75].

The results one day post-radiation supports a generally accepted presumption that the biological system responds differently to chronic low and acute high dose rates when given the same total dose. However, this statement is highly debated due to inconsistent cellular, animal, and human research results [40,76]. The dose rate related response, seen in this study, could be linked to several factors like type and repair of DNA damage [77], as the DNA damage response introduces alterations to the chromatin structure (reviewed in [78]). Changes in the chromatin packaging are also suggested to be an essential factor for DNA damage, as condensed chromatin, due to the nucleosome-binding of DNA [79], is assumed to be more resistant to radiation damage and the attack from ROS [80–82]. Genomic regions depleted of nucleosomes, such as promoters, are thus more susceptible to DNA damage. This could result in restricting the genomic distribution of possible DNA damage sites. Therefore, we hypothesize that the difference in chromatin accessibility could be due to the difference in the genotoxic susceptibility between the chronic LDR and acute HDR. However, as the open chromatin regions in tissue represent an average accessibility profile generated from heterogeneous cell types and chromatin states, we anticipate that chromatin changes related to DNA damage-sensing and -repair cannot be detected in bulk tissue using ATAC-Seq alone.

The long-term changes in chromatin accessibility demonstrated a clear dose-rate-specific response. Interestingly, only a long-term response after HDR exposure was identified, while no measurable changes were seen after the LDR irradiation. After the HDR exposure, a significant differential reduction in accessibility was inflicted, primarily confined to transcriptional start sites (TSS). The magnitude of the late DARs was in the same order as HDR_early, but the regions did not overlap. Additionally, only a few DAR-associated genes (DAGs) overlapped between the two HDR time points. Nevertheless, the enrichment analysis revealed common biological pathways at the two time points. Suggesting that different regions could be connected via biological functions, even if the overlap between the DARs and the DAGs were marginal. Taken together, a shift in chromatin conformation in the period between the sampling timepoints is evident, where the chromatin state after the low dose rate is restored whilst the high dose rate induces long-term changes.

The biological terms enriched for HDR_late are related to known radiation-induced effects, such as DNA double-strand breaks. Other interesting pathways included transcription factor binding relevant for RNA polymerase II, the AP-1 complex and the transcription repressor complex, terms sharing the transcription factors *Jun* and *Fos*. These genes are well-characterized as immediate-early response genes (IEGs) [83], radiation-responsive proto-oncogenes [84], and also, they are the two dimers constituting the AP-1 complex [85]. As we report a reduction in accessibility over TSS in genes enriched to these critical processes, we hypothesize that these results could be related to molecular processes hindering the transposase from accessing the DNA [86,87]. Such could include the protein binding to the DNA (e.g., TFs and polymerase II) [33] or changes in epigenetic states. Identifying specific protein-binding motifs within the DARs genomic sequence could enlighten this issue and should be pursued in future studies.

Epigenetic mechanisms involving altered chromatin accessibility have been linked to multiple radiation-induced effects (reviewed in [28,88]) like histone-methylation (H3K27me3 [89]) resulting in gene repression or gene-specific hyper-methylation [90]. Such changes could be a result of exposure-induced poised or primed genes/promoters. Primed/poised genes are transcriptionally silenced genes in the absence of stimulus, but the promoters have both repressive and activating properties for rapid activation upon new stimuli [91]. It is thus interesting to note that we observed reduced accessibility of enriched terms related to transcriptional activity. A genome-wide mapping study of chromatin states identified repressed TSSs enriched with active chromatin marks and RNA polymerase II. They also showed that repressive and activating properties are strongly associated with IEGs [92]. A repressed chromatin state does not necessarily represent a condensed chromatin state that hinders transcription but, in contrast, represents a regulatory mark for rapid transcriptional activation upon subsequent stimuli [93]. Taken together, poised/primed genes could be a plausible explanation for our results that could represent essential mechanisms in radiation-induced adaptive response. However, this notion must be verified by methods commentary to ATAC-Seq.

Other factors for the observed long-term HDR response could be linked to changes in the cellular composition of the liver due to cell death, differentiation, or senescence. However, the abovementioned liver responses are seen related to the doses and dose rate used [28,94]. The bulk liver tissue also contains multiple cell types, each of which could have distinct epigenomic patterns. Without commentary methods, it is challenging to comprehend the extent of these possible mechanisms and whether they appear to the extent that would affect the overall ATAC-Seq output. However, hepatocytes are the dominant cell type in the liver [95], and when we compared the gene expression profile [36] with single-cell RNA-Seq data from flow cytometry separated mouse liver cells, the gene expression profile was calculated to comprise >98% hepatocytes (data not shown). We, therefore, assume that the hepatocyte epigenome dominates the signal in these datasets. To detect long-term effects after chronic low dose rate exposure may require a larger experiment than this current study. However, the HDR_late results are evident despite inherent experimental factors (like biological replicates and bulk tissue samples), highlighting a critical difference in acute and chronic long-term potential even though the total dose is the same.

## Conclusions

To our knowledge, this is the first study to evaluate the impact of ionizing radiation low chronic vs high acute dose rate exposure on chromatin accessibility to identical total dose of 3 Gy. We show that chronic low dose rate exposure to a high total dose of 3 Gy do not induce permanent changes in chromatin accessibility, in contrast to the acutely given high dose rate of the same total dose where repressed promoter regions in genes relevant for DNA damage and transcriptional regulation were evident. Our results highlight that dose rate and exposure regime are relevant factors for radiation-induced epigenomic changes to mice liver and is important for understanding long-term changes after ionizing radiation exposure. However, as the ATAC-Seq method alone is insufficient to capture the mechanisms leading to the observed results, future studies are needed to fully comprehend the biological consequence of these findings.

## Supplementary Material

Supplemental Material

## Data Availability

The data that support the findings of this study are openly available in the Sequence Read Archive (SRA) at https://www.ncbi.nlm.nih.gov/sra, with the reference nr: PRJNA832920.
