# Supplementary material for: Dose rate dependent reduction in chromatin accessibility at transcriptional start sites long time after exposure to gamma radiation
Source: Epigenetics. 2023 Mar 27;18(1):2193936. doi: 10.1080/15592294.2023.2193936 (PMC10054331; doi:10.1080/15592294.2023.2193936)
Supplement: Supplemental Material [file KEPI_A_2193936_SM7812.zip › Supplementary files/Supplementary legends.docx]

# Appendices (as appropriate);

**Supplementary 1 (S1)**

Details of the bioinformatic pipeline used for data pre-processing (1-3) and downstream analysis (4-5).

**Supplementary 2 (S2)**

The complete MetaScape-output.

**Supplementary 3 (S3)**

ATAC-Seq metadata and mapping statistics.

**Supplementary 4 (S4)**

*The fragment distribution per sample in S4_QC_library_trace_allsample.zip*
