# Supplementary material for: Dose rate dependent reduction in chromatin accessibility at transcriptional start sites long time after exposure to gamma radiation
Source: Epigenetics. 2023 Mar 27;18(1):2193936. doi: 10.1080/15592294.2023.2193936 (PMC10054331; doi:10.1080/15592294.2023.2193936)
Supplement: Supplemental Material [file KEPI_A_2193936_SM7812.zip › Supplementary files/Supplementary_1.docx]

**Supplementary 1 (S1)** Details of the pipeline used for data pre-processing (1-3) and downstream analysis (4-5).

1. nf-core ATAC-seq pipeline

The detailed pipeline command used in the pre-processing analysis can be found under the following GitHub repository: https://github.com/nf-core/atacseq

Script name used: main.nf

Workflow container version: nfcore/atacseq:1.2.1

Container engine: Singularity v3.6.4

Nextflow version: version 20.11.0-edge, build 5448 (16-11-2020 08:23 UTC)

Workflow summary of the ATAC-seq pipeline contained following arguments:

-profile singularity,

--genome GRCm38,

--input design.csv,

--max_cpus 8.

Workflow was run with maximal resources: 31 GB memory, 8 CPU, 10 days per job given.

1. Command for extraction of nucleosome free regions from sorted .bam-files

samtools view -h “input” | perl -lane '$l = 0; $F[5] =~ s/(\d+)[MX=DN]/$l+=$1/eg; print if $l < 100 or /^@/' | samtools view -bS - > “output”

1. Peak Calling with MACS2 v2.2.7

macs2 callpeak -t “input” -f BAMPE --outdir ./ --name “output” -g mm

1. Differential analysis with DESeq2 (data post processing)

dds <- DESeqDataSetFromMatrix(countData, colData, ~group + age, rowRanges = consensusToCount)

dds <- DESeq(dds)

1. Annotation of contrasts with annotatePeak package and TxDb

contrastData_annotated <- annotatePeak(contrastData, TxDb = TxDb.Mmusculus.UCSC.mm10.knownGene)
