# Supplementary material for: Dose rate dependent reduction in chromatin accessibility at transcriptional start sites long time after exposure to gamma radiation
Source: Epigenetics. 2023 Mar 27;18(1):2193936. doi: 10.1080/15592294.2023.2193936 (PMC10054331; doi:10.1080/15592294.2023.2193936)
Supplement: Supplemental Material [file KEPI_A_2193936_SM7812.zip › Supplementary files/Updated Supplemetary_3.docx]

**Supplementary Table 1. ATAC-Seq metadata and mapping statistics.**

| **Group** | **Sample ID** | **QC-passed Reads** | **Mapped (%)** | **Properly Paired (%)** | **NFR Reads (<100bp)** | **NFR Reads (%)** | **QC-passed Peaks** | **Reads In Peaks (RIP) (%)** |
| --- | --- | --- | --- | --- | --- | --- | --- | --- |
| CTRL | R1 | 126,340,392 | 99.20 | 97.46 | 31,602,553 | 25.67 | 84,668 | 22.90 |
| CTRL | R2 | 101,390,920 | 99.24 | 98.16 | 16,341,608 | 16.42 | 33,834 | 9.43 |
| CTRL | R3 | 108,434,882 | 99.46 | 96.98 | 26,904,111 | 25.58 | 71,562 | 17.70 |
| CTRL | R4 | 119,045,374 | 99.60 | 98.57 | 23,415,170 | 19.95 | 61,287 | 16.50 |
| CTRL | R5 | 101,809,468 | 99.24 | 96.58 | 27,211,347 | 27.67 | 90,235 | 25.60 |
| LDR | R1 | 109,019,826 | 98.98 | 98.01 | 18,260,419 | 17.09 | 33,511 | 8.32 |
| LDR | R2 | 119,542,410 | 99.19 | 98.21 | 23,936,039 | 20.39 | 85,433 | 29.60 |
| LDR | R3 | 139,725,676 | 99.33 | 98.38 | 27,009,957 | 19.65 | 57,127 | 12.30 |
| MDR | R1 | 104,344,204 | 96.91 | 95.80 | 19,587,762 | 19.60 | 40,734 | 10.50 |
| MDR | R2 | 112,859,786 | 98.91 | 97.74 | 19,324,976 | 17.52 | 55,206 | 18.10 |
| MDR | R3 | 123,694,052 | 99.49 | 98.24 | 21,694,519 | 17.85 | 41,636 | 9.75 |
| HDR | R1 | 112,942,896 | 99.54 | 97.51 | 30,931,600 | 28.09 | 84,327 | 22.80 |
| HDR | R2 | 103,929,930 | 99.45 | 97.39 | 27,856,183 | 27.52 | 85,469 | 26.10 |
| HDR | R3 | 134,222,048 | 99.36 | 98.10 | 29,457,900 | 22.37 | 78,373 | 19.80 |
| CTRL_late | R1 | 102,700,534 | 98.51 | 97.37 | 19,084,020 | 19.08 | 38,504 | 9.89 |
| CTRL_late | R2 | 118,384,442 | 98.82 | 97.67 | 20,308,738 | 17.56 | 68,267 | 21.60 |
| CTRL_late | R3 | 112,638,514 | 98.58 | 97.55 | 21,380,994 | 19.46 | 39,718 | 9.34 |
| CTRL_late | R4 | 111,220,712 | 97.65 | 96.42 | 32,469,830 | 30.28 | 83,292 | 19.80 |
| LDR_late | R1 | 105,383,454 | 98.96 | 97.94 | 23,046,990 | 22.33 | 26,057 | 5.07 |
| LDR_late | R2 | 132,908,782 | 95.16 | 93.84 | 25,580,042 | 20.51 | 74,029 | 20.50 |
| LDR_late | R3 | 114,117,356 | 99.06 | 97.97 | 24,964,040 | 22.33 | 37,179 | 7.28 |
| HDR_late | R1 | 107,317,296 | 98.07 | 95.54 | 36,590,080 | 35.69 | 20,123 | 2.44 |
| HDR_late | R2 | 102,548,600 | 96.51 | 95.46 | 17,975,665 | 18.36 | 67,396 | 23.40 |
| HDR_late | R3 | 120,073,874 | 99.13 | 98.12 | 21,856,440 | 18.55 | 29,440 | 5.53 |
